# Characteristics of summer hourly precipitation under different urbanization background in central China

**DOI:** 10.1038/s41598-022-11487-z

**Published:** 2022-05-09

**Authors:** Yonglan Tang, Guirong Xu, Rong Wan, Xiaofang Wang

**Affiliations:** Hubei Key Laboratory for Heavy Rain Monitoring and Warning Research, Institute of Heavy Rain, China Meteorological Administration, Wuhan, 430205 China

**Keywords:** Climate sciences, Environmental sciences, Natural hazards

## Abstract

The relationship between sub-daily precipitation and urbanization is widely concerned because short-term precipitation is sensitive to urbanization and difficult to predict. Using the data of summer hourly precipitation and urban development during 2007–2019 at four urban stations and an atmospheric background monitoring station in central China, this study investigates the characteristics of hourly precipitation and hourly extreme precipitation (HEP) under different urbanization background. It is found that high urbanization level may benefit precipitation intensity but not for accumulated precipitation amount and precipitation frequency, and it is also conducive to the occurrence of hourly precipitation within [20, 50) mm. Precipitation amount and frequency for hourly precipitation within [5, 50) mm have similar diurnal variation at fixed station, yet the diurnal variation of precipitation intensity is insignificant. The differences in temporal variation of precipitation are related to urbanization and terrain. Both high urbanization level and speed are conducive to summer HEP; especially summer HEP intensity may increase gradually under sustainable urbanization development. Although growth-type HEP occurs frequently with main contribution to total HEP precipitation amount in central China regardless of urbanization level, the frequency and contribution of continuous-type HEP tends to increase under high urbanization level and speed.

## Introduction

With the development of urbanization, human activities and urban construction have changed the urban underlying surface and meteorological environment, which in turn cause the changes in weather, climate and atmospheric environment. Many studies have found that urbanization leads to the increase of urban precipitation amount and heavy precipitation events, and the damage of precipitation-induced flooding is serious^1–7^. Based on ground rain gauge data, Shepherd and Burian^8^ found that urban heat island effect (UHIE) of Houston city of USA had impact on urban precipitation anomaly. The climate characteristics of urban precipitation in India show that the probability of heavy precipitation in urban area is much higher than that in non-urban area, and the increasing trend of extreme precipitation events in urban area is more obvious^9^. Using daily precipitation, temperature and population data, Vargas and Magaña^10^ found that the rapid development of Mexico city metropolitan area led to the development of strong urban heat island (UHI) and the increase of natural disasters related to rainstorms (precipitation > 20 mm/day). In China, Liang et al.^11^ investigated the spatial difference of precipitation in Shanghai during the periods of slow and rapid urbanization, and they indicated that the spatial distribution of annual precipitation amount and rainstorm frequency exhibited obvious urban “rain island” characteristics. The above studies show that the urbanization plays an important role in urban climate especially urban precipitation all over the world.

Many observational statistics and numerical simulation indicate that the precipitation on sub-diurnal scale is more sensitive to urbanization and climate warming than that on other scales, and temperature rise makes heavy precipitation tend to occur in a shorter time (up to a few hours), also the increase rate of heavy precipitation on sub-diurnal scale is higher than that on diurnal scale^12–15^. Compared to heavy precipitation caused by long-term accumulation, the heavy precipitation on sub-diurnal scale has the characteristics of small spatial scale, rapid development and great difficulty in prediction, which poses a great challenge to meteorological operational forecast, urban construction and social emergency management. Therefore, the relationship between precipitation on sub-diurnal scale and urbanization has been widely concerned. Changnon^16^ indicated that the heat island circulation can trigger and enhance convective weather, and also change the dynamic structure of clouds and storms as well as the urban precipitation on sub-diurnal scale. With the analysis on six precipitation events in Atlanta of USA, Bornstein and Lin^17^ found that the UHI induced a convergence area and then caused three storms at different times of the day. Similar studies are also emerging in China. Zhang et al.^2^ and Yuan et al.^18^ suggested that the strong UHIE of Beijing-Tianjin-Hebei cities could excite or strengthen convective system, which is conducive to the occurrence of short-term heavy precipitation. According to Yang et al.^19^, the short-term heavy precipitation events tend to occur in or near the central urban area of Beijing and are related with the UHI intensity. Liang and Ding^6^ indicated that the frequency and amount of annual hourly extreme precipitation (HEP) in Shanghai increased since 1980 especially in the center urban area. According to Wang et al.^7^, in the urbanization process of the Yangtze River Delta, the HEP tends to occur in urban area. Luo et al.^20^ found that in the Pearl River Delta urban agglomeration, the frequency of HEP increased and the strong UHIE enhanced the hourly precipitation generated by both local convective system and that moving in from outside. These studies explore the impact of urbanization on short-term precipitation and enrich the knowledge of short-term precipitation and its relationship with urbanization. However, these studies in China are with little consideration on the influence of terrain and precipitation grading. Due to the complex influence of urbanization on precipitation, it is necessary to study the characteristics of hourly precipitation at various grades under different urbanization background combined with the variety of terrain.

Atmospheric background monitoring station (ABMS) is deployed globally by the World Meteorological Organization (WMO) to carry out various observations including greenhouse gases, atmospheric ozone, aerosols, solar radiation, precipitation chemistry and boundary layer meteorology, under the global reference atmospheric background conditions. Currently, the observation of ABMS is mostly adopted to study the changes of atmospheric components, such as aerosols and air pollutants, and their long-distance transport characteristics^21–24^, but rarely used to study the precipitation characteristics at ABMS. Before 2020, there are only seven ABMSs in China and only one ABMS in central China. In this study, using the data of summer hourly precipitation and urban development at four urban stations and an ABMS in central China from 2007 to 2019, the interannual and diurnal variations of summer precipitation and the characteristics of HEP are analyzed, and the precipitation difference at various grades as well as under different urbanization background is also investigated with consideration of terrain.

## Data and methods

**Figure 1:**
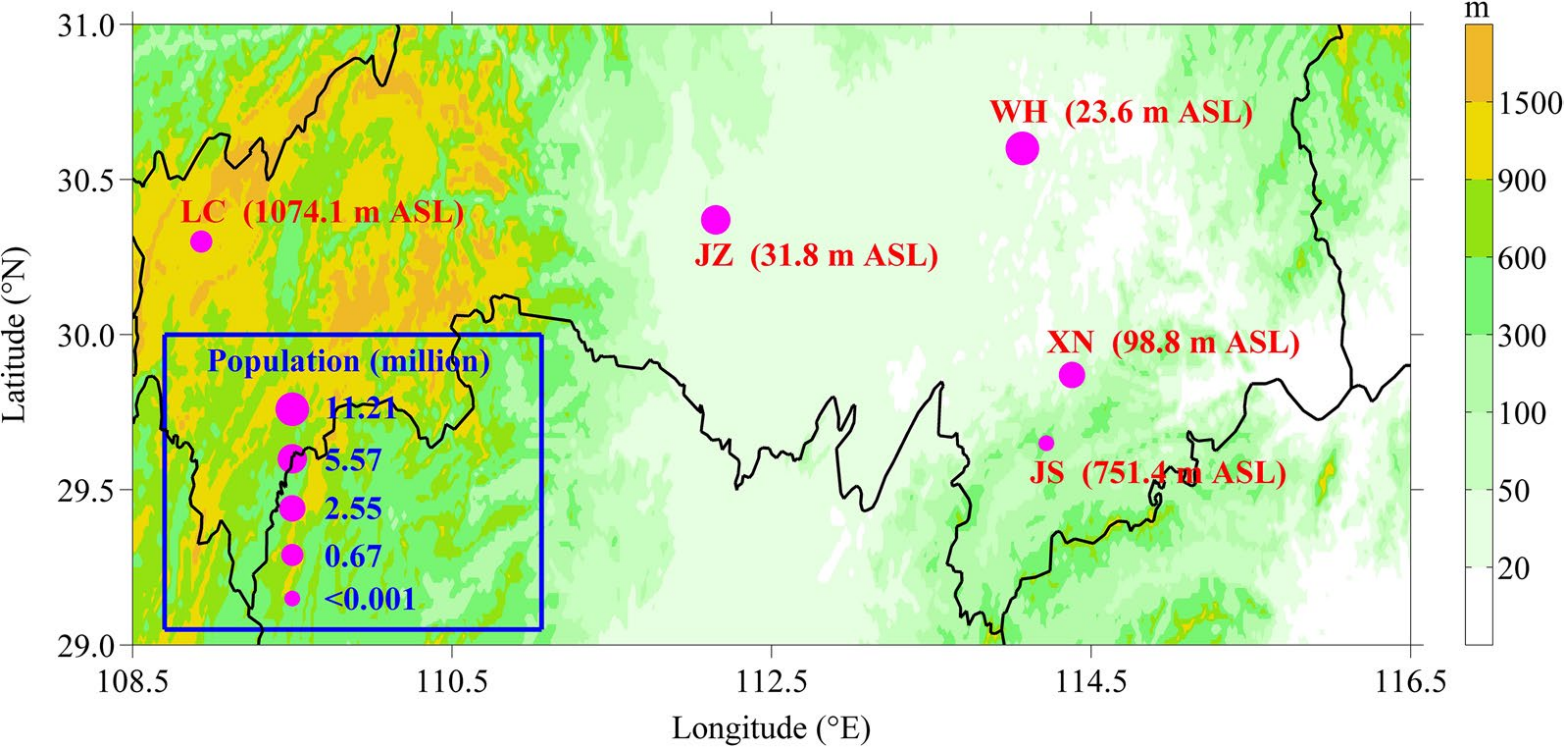
Geographic location of Wuhan (WH), Jingzhou (JZ), Xianning (XN), Lichuan (LC) and Jingsha (JS) stations in central China and their population in 2019. This map is generated with the MATLAB software [Version: 8.3.0.532 (R2014a)] of the MathWorks Incorporation (http://www.mathworks.com/), and the terrain data comes from the ETOPO1 Bedrock data provided by the National Geophysical Data Center (NGDC) of the United States (http://www.ngdc.noaa.gov/mgg/global/etopo1source.html).

Four urban stations and an ABMS in central China are focused in this study. These five stations are located in the region with latitude range of 29.6°–30.6°N and longitude range of 108.9°–114.4°E but with various urbanization level and terrain. As shown in Fig. 1, Wuhan (WH), Jingzhou (JZ), Xianning (XN) and Lichuan (LC) have a population of 11.21, 5.57, 2.55 and 0.67 million in 2019 respectively, while Jingsha (JS) is an ABMS far away from urbanization area. As an ABMS, JS is located in small mountain area with altitude of 751.4 m above sea level (ASL), while LC is located in large mountain area with altitude of 1074.1 m ASL, XN is located to small hills with altitude of 98.8 m ASL, JZ and WH are located in plain with altitude of 31.8 and 23.6 m ASL, respectively. The station geographic information and summer (June–August) hourly precipitation data of these five stations are obtained from the National Meteorological Information Center of China (http://data.cma.cn/), and the urban development data including the population and urban built-up area are from the Hubei Provincial Bureau of Statistics (http://tjj.hubei.gov.cn/) and the Urban Construction Year Book (http://www.mohurd.gov.cn/). As the urban development data before 2007 is not available, this study just focuses on the period of 2007–2019. In addition, the urban boundary data comes from the satellite-based land use category maps supported by Tsinghua University of China (http://data.ess.tsinghua.edu.cn).

**Figure 2:**
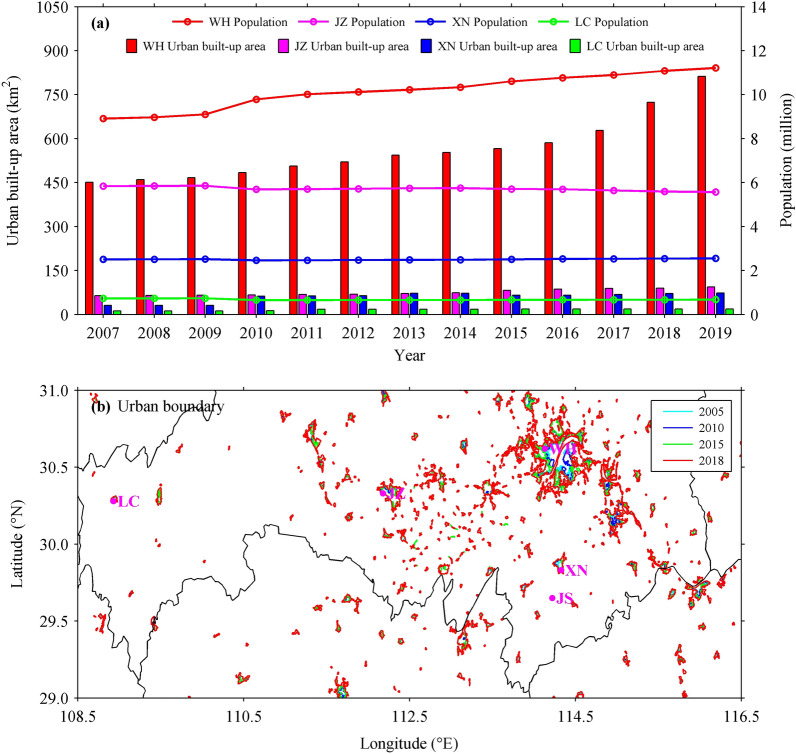
(**a**) Interannual variations of the population and urban built-up area in WH, JZ, XN and LC cities during 2007–2019, and (**b**) urban boundaries in study region during 2005–2018 obtained from the satellite-based land use category maps (http://data.ess.tsinghua.edu.cn).

In the urbanization process, the population continues to gather to the city, and the city scale continues to expand. Therefore, the rapid urbanization is usually accompanied by the rapid growths of population and urban built-up area, which are generally taken as indicators to reflect the urbanization process^25,26^. Figure 2a presents the interannual variations of the population and urban built-up area in WH, JZ, XN and LC cities during 2007–2019. The population and urban built-up area of WH are the highest and show an increasing trend. Since 2010, WH has a population above 10 million while the other cities have a relatively stable population less than 6 million, also the urban built-up area of WH has exceeded 500 km^2^ while those of the other cities are less than 100 km^2^. By 2019, the urban built-up areas of WH, JZ, XN and LC are 812.39, 93.87, 73.25 and 19.00 km^2^, respectively. According to the specifications and standards of the WMO, ABMS is set up in the area far away from urbanization, that is to say, the urbanization process of ABMS is almost zero. As shown in Fig. 2b, the urban boundaries, which are generated from the 30-m global artificial impervious area data^27,28^, well delineate the urban extents of WH, JZ, XN, LC and JS stations during 2005–2018, and show a good agreement with the results derived from urban development data. Therefore, WH has the fastest urbanization process, followed by JZ, XN and LC, and JS, as an ABMS, has none urbanization. As urban meteorological events are influenced by human activity, climate change and other factors^20,26^, and this study mainly focuses on the impact of urbanization, so WH, XN and JS are used to investigate the characteristics of summer hourly precipitation under different urbanization background. This is because JS is an ABMS, which monitors the atmospheric natural change under none urbanization in central China, while WH and XN have significantly different urbanization levels, especially WH and XN are close to JS, which may reduce the impact of regional climate differences. The other two stations of JZ and LC are analyzed for comparison in discussing on the results.

Due to lack of comparability to define HEP events by absolute threshold especially for different terrains, a threshold test method is used to define HEP with a certain percentile value^29,30^. In practice, for each station, the hourly precipitation beyond 0.1 mm in the study period is arranged in ascending order, and the value at a certain percentage is selected as the threshold of HEP at the station. If the hourly precipitation exceeds the threshold, it is considered that a HEP event occurs. The HEP frequency is defined as the number of HEP occurrence, while the HEP amount is defined as the cumulative precipitation amount of HEP events, and the HEP intensity is the ratio of HEP amount to HEP frequency.

According to the existing studies^31–33^, seven hourly precipitation criteria are defined, namely 0.1 mm, 0.5 mm, 1 mm, 5 mm, 10 mm, 20 mm, 50 mm, to classify hourly precipitation into various grades. Moreover, referring to the definition method of the Central Meteorological Observatory of China, the hourly precipitation greater than or equal to 20.0 mm is defined as short-term heavy precipitation, and the hourly precipitation greater than or equal to 50.0 mm is defined as short-term extremely heavy precipitation^34^.

In the study on diurnal variation, for each hour, the precipitation amount is accumulated by all the hourly precipitation amount in this hour during the entire study period, in the same way, the precipitation frequency is the number of all the hourly precipitation events in this hour, and the precipitation intensity is the ratio of precipitation amount to precipitation frequency in this hour. After that, according to the methods of Li et al.^35^ and Yu and Li^36^, the precipitation amount, frequency and intensity in each hour are divided by their mean values in 1–24 h of the day to obtain the dimensionless diurnal variation series of these parameters. This dimensionless processing allows the diurnal variation of precipitation amount, frequency and intensity to be displayed in the same vertical coordinate, which is convenient for comparative analysis. In addition, the least square method is used in the study on interannual variation to find a one-variable linear equation of the trend change of meteorological elements.

## Results

### Interannual variation of summer hourly precipitation

**Figure 3:**
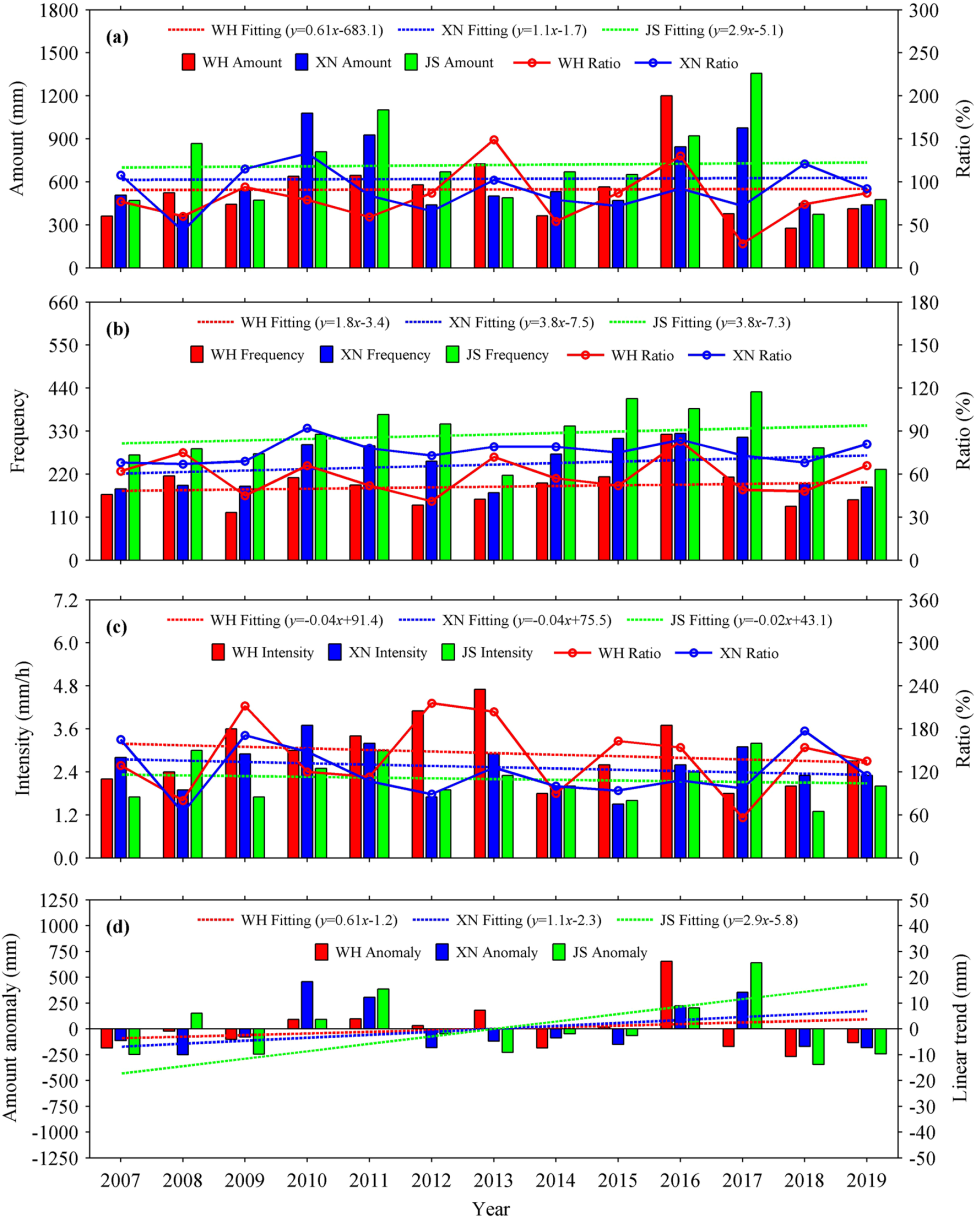
Interannual variations of (**a**) accumulated precipitation amount, (**b**) precipitation frequency, (**c**) precipitation intensity and (**d**) accumulated precipitation amount anomaly obtained from the summer hourly precipitation data of WH, XN and JS stations during 2007–2019. The ratio of precipitation parameter between urban stations and JS is shown in the right axis in (**a**–**c**), and the linear trend of accumulated precipitation amount anomaly is shown in the right axis in (**d**).

Figure 3 presents the interannual variations of accumulated precipitation amount, precipitation frequency, precipitation intensity and accumulated precipitation amount anomaly obtained from the summer hourly precipitation data of WH, XN and JS during 2007–2019. The summer accumulated precipitation amounts of the three stations show weak linear increasing trend. WH has the smallest linear slope of 0.61 mm/a and JS has the largest one of 2.9 mm/a, while XN has a linear slope of 1.1 mm/a; moreover, the summer accumulated precipitation amount is the largest at JS, followed by XN, and the smallest at WH in most years. It is the same situation for the summer precipitation frequency. Although WH has the smallest linear slope of precipitation frequency (1.8/a), XN and JS have an equal linear slope (3.8/a). However, all the summer precipitation intensities of WH, XN and JS show weak decreasing trend during 2007–2019, with corresponding linear slopes of − 0.04, − 0.04 and − 0.02 mm/h per year. In general, the summer precipitation intensity is the highest at WH, followed by XN, and the lowest in JS, which is opposite to those for accumulated precipitation amount and precipitation frequency. The maximum precipitation intensities of WH, XN and JS are 4.7, 3.7 and 3.2 mm/h, respectively, increasing with urbanization level. Additionally, because JS is far away from urbanization area, thus the ratio of precipitation parameter between urban station and JS can partly reflect the urbanization effect on precipitation parameter. As shown in Fig. 3a–c, the ratios of accumulated precipitation amount, precipitation frequency and precipitation intensity between WH and JS vary in ranges of 28–149%, 41–83% and 56–216%, respectively, and these ranges are 43%-133%, 67–92% and 63–177% between XN and JS, showing large range in urban station with high urbanization level. Besides, in order to remove the climate background impacts on precipitation, the annual time series of accumulated precipitation amount is subtracted by its mean value to obtain the interannual variation of accumulated precipitation amount anomaly. Figure 3d shows that the interannual variations of accumulated precipitation amount anomaly at WH, XN and JS also present weak linear increasing trend, with linear slopes of 0.61, 1.1 and 2.9 mm/a, respectively, and this is consistent with the results derived from accumulated precipitation amount. These results indicate that the accumulated precipitation amount and precipitation frequency of summer hourly precipitation in central China show weak linear increasing trend during 2007–2019, while the precipitation intensity of summer hourly precipitation shows weak linear decreasing trend; moreover, the higher the urbanization level, the lower the accumulated precipitation amount and precipitation frequency, but the higher the precipitation intensity. Note that the urbanization level of WH is rising during 2007–2019 (Fig. 1), while its precipitation intensity shows a downward trend (Fig. 3c). This is because urban precipitation is a comprehensive reflection of many influencing factors, in addition to urbanization, it is also affected by climate change, terrain and so on^20^. As shown in Fig. 3c, the precipitation intensity of JS is decreasing during 2007–2019, indicating a downward trend under climate change background, and the climate change also result in the same trend on the precipitation intensities of WH and XN. However, Fig. 3c also shows that in the same period, the precipitation intensity is higher at the station with higher urbanization level.

**Figure 4:**
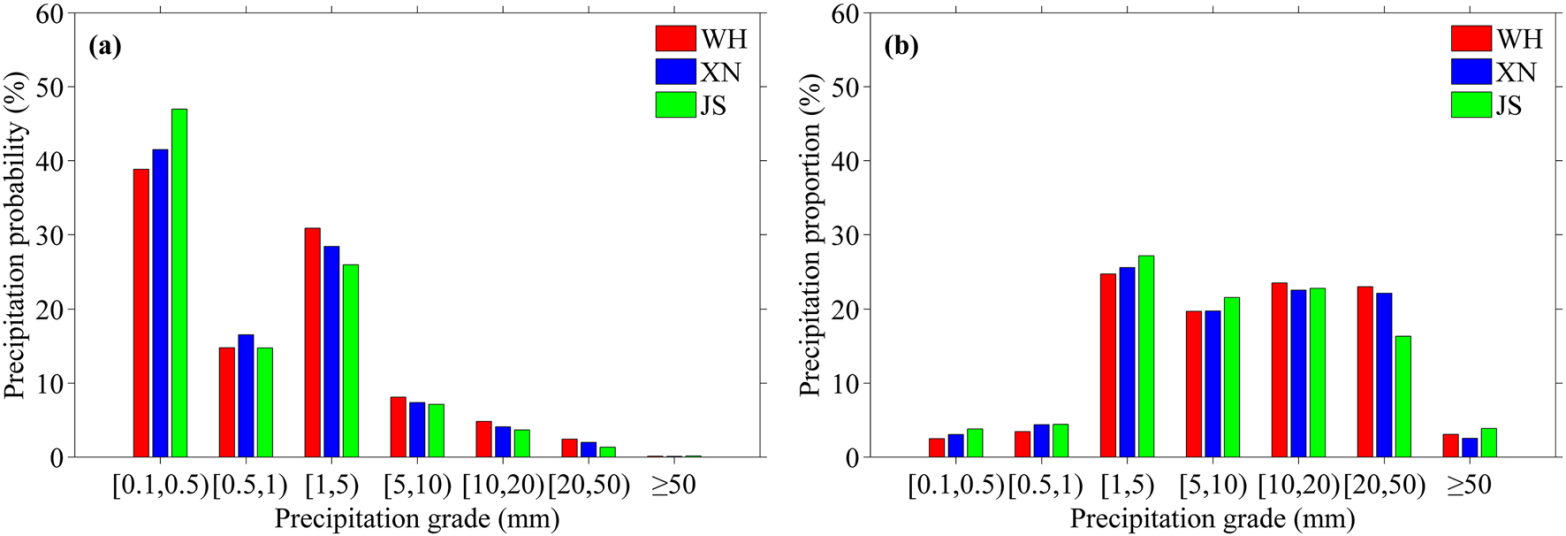
Precipitation (**a**) probability and (**b**) proportion in different grades obtained from summer hourly precipitation data of WH, XN and JS stations during 2007–2019.

To further investigate the relationship between summer hourly precipitation and urbanization, the precipitation probability and proportion in different grades obtained from summer hourly precipitation at WH, XN and JZ during 2007–2019 are presented in Fig. 4. Note that precipitation probability is the ratio of precipitation frequency in a certain grade (or type) to total precipitation frequency, and precipitation proportion is the ratio of precipitation amount in a certain grade (or type) to total precipitation amount. As shown in Fig. 4a, when hourly precipitation is in the four grades of [1, 5) mm, [5, 10) mm, [10, 20) mm and [20, 50) mm, the precipitation probabilities of the three stations are ordered as WH > XN > JS, indicating that the precipitation probability of hourly precipitation within [1, 50) mm increases with urbanization level. Moreover, the precipitation proportion of hourly precipitation within [20, 50) mm also increases with urbanization level, but it is opposite for hourly precipitation less than 10 mm (see Fig. 4b). It can be concluded that urbanization is conducive to the occurrence of hourly strong precipitation, and this is consistent with the conclusion of Liang and Ding^6^. Furthermore, for hourly precipitation greater than or equal to 50 mm, the precipitation probabilities at WH, XN and JS are 0.12%, 0.09% and 0.14%, respectively, and those for precipitation proportion are 3.07%, 2.54% and 3.86%, indicating that short-term extremely heavy precipitation tends to be stronger in mountain area. This is because when the unstable warm moist flow passes through the mountains, it is strengthened due to the effect of terrain and this is comfortable for triggering short-term extremely heavy precipitation^37–39^.

### Diurnal variation of summer hourly precipitation

**Figure 5:**
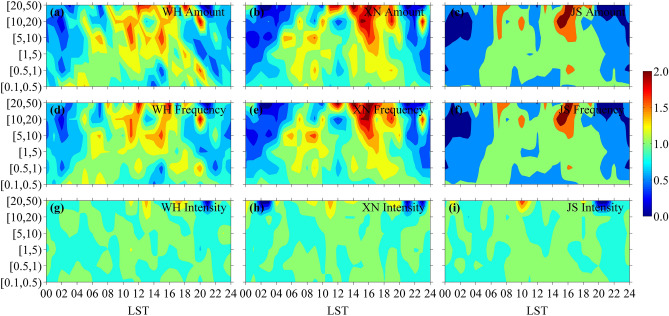
Diurnal variations of precipitation (**a**–**c**) amount, (**d**–**f**) frequency and (**g**–**i**) intensity with dimensionless processing obtained from summer hourly precipitation data of WH (left), XN (middle) and JS (right) stations during 2007–2019.

Some studies suggest that the change of temporal distribution pattern of precipitation caused by urbanization is an important embodiment of urbanization effect, and the most direct reflection is the influence of urbanization on diurnal variation of precipitation^3,40–42^. Due to the small sample number of short-term extremely heavy precipitation, which is defined as the hourly precipitation greater than or equal to 50 mm, it is not included in the study of diurnal variation of summer hourly precipitation. As shown in Fig. 5a–c, the diurnal variation pattern of precipitation amount varies with urbanization level for hourly precipitation in grades of [5, 10) mm, [10, 20) mm and [20, 50) mm. At WH, the diurnal variation of precipitation amount tends to be bimodal, with two equivalent peaks in 10–12 LST and 14–16 LST, respectively. While at XN, the diurnal variation of precipitation amount tends to be unimodal with high values in 14–18 LST. Although the diurnal variation of precipitation amount at JS tends to be bimodal, the main peak in 14–18 LST is evidently higher than the secondary peak in 07–08 LST. As the terrains of WH, XN and JS are different, the different patterns of precipitation amount at the three stations may also be influenced by the terrain, and this suggests that urban precipitation is closely related to urbanization and terrain, which is a comprehensive performance of various influence factors^7,43^. Moreover, the diurnal variations of precipitation frequency at the three stations are the same situation, because precipitation frequency presents a similar pattern of diurnal variation to that for precipitation amount at the same station (see Fig. 5d–f). However, the diurnal variation of precipitation intensity at the three stations are not significant, yet for hourly precipitation in grade of [20, 50) mm, the daily variation range of precipitation intensity at JS is larger than those at WH and XN (see Fig. 5g–i). This is because JS is located in mountain area, where short-term heavy precipitation is easily triggered and strengthened due to the effect of terrain^37–39^. Apparently, there is difference in diurnal variation of hourly precipitation at the three stations with different terrain, indicating the impact of terrain on the diurnal variation of hourly precipitation^35^. Besides the terrain, different urbanization levels may also lead to difference in diurnal variation of hourly precipitation. The faster the urbanization process is, the stronger the UHIE is, and this intensifies the local convective movement in the city, also there are more condensates in the city, thus the mechanism of urban precipitation is more complex^44,45^. Therefore, urban precipitation tends to occur more sudden and random under higher urbanization level, showing more fluctuation in its sub-diurnal variation, and this may explain that the diurnal variation pattern of hourly precipitation at WH is more complex than that at JS while XN is in between. This also supports the conclusion of Niyogi et al.^42^ that precipitation time is more dispersed with the expansion of the city. It should be noted that the diurnal variations of precipitation amount and frequency at JS is evidently different from those at WH and XN. This is because JS is an ABMS, which monitors the atmospheric environment not affected by human activity according to the specifications and standards of the WMO, and the changes of precipitation amount and frequency at JS reflect their own natural variability; while WH and XN are in different urbanization process, and their precipitation and frequency changes are affected by natural variability and urbanization. Therefore, the difference in diurnal variations of precipitation amount and frequency at WH, XN and JS can partly reflect the impact of urbanization.

**Figure 6:**
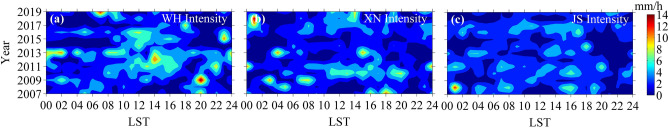
Diurnal variations of precipitation intensity obtained from summer hourly precipitation data of (**a**) WH, (**b**) XN and (**c**) JS stations in each year of 2007–2019.

Figure 6 presents the diurnal variations of summer precipitation intensity at WH, XN and JS stations in each year of 2007–2019. The interannual differences of diurnal variation of summer precipitation intensity at the three stations are ordered as WH > XN > JS, indicating that the interannual difference of diurnal variation increases with urbanization level. As an ABMS, JS monitors the average state of atmospheric background environment. Figure 6c shows that the diurnal variation of summer precipitation intensity at JS presents small interannual difference, and this reflects the stability of atmospheric background environment. Compared to JS, the diurnal variations of summer precipitation intensity at XN and WH present larger interannual difference. Different from ABMS, the atmospheric composition over the city is complex due to urbanization, which, coupled with the friction of urban underlying surface, the block of buildings, thermal turbulence over urban area and other influence factors, makes the mechanism of urban precipitation also complicated^44,45^, and this may be partly reflected by the interannual difference of precipitation characteristics. Hence, the interannual difference of diurnal variation of summer precipitation intensity at urban station is larger than that at ABMS station and increases with urbanization level.

### Characteristics of summer HEP

**Figure 7:**
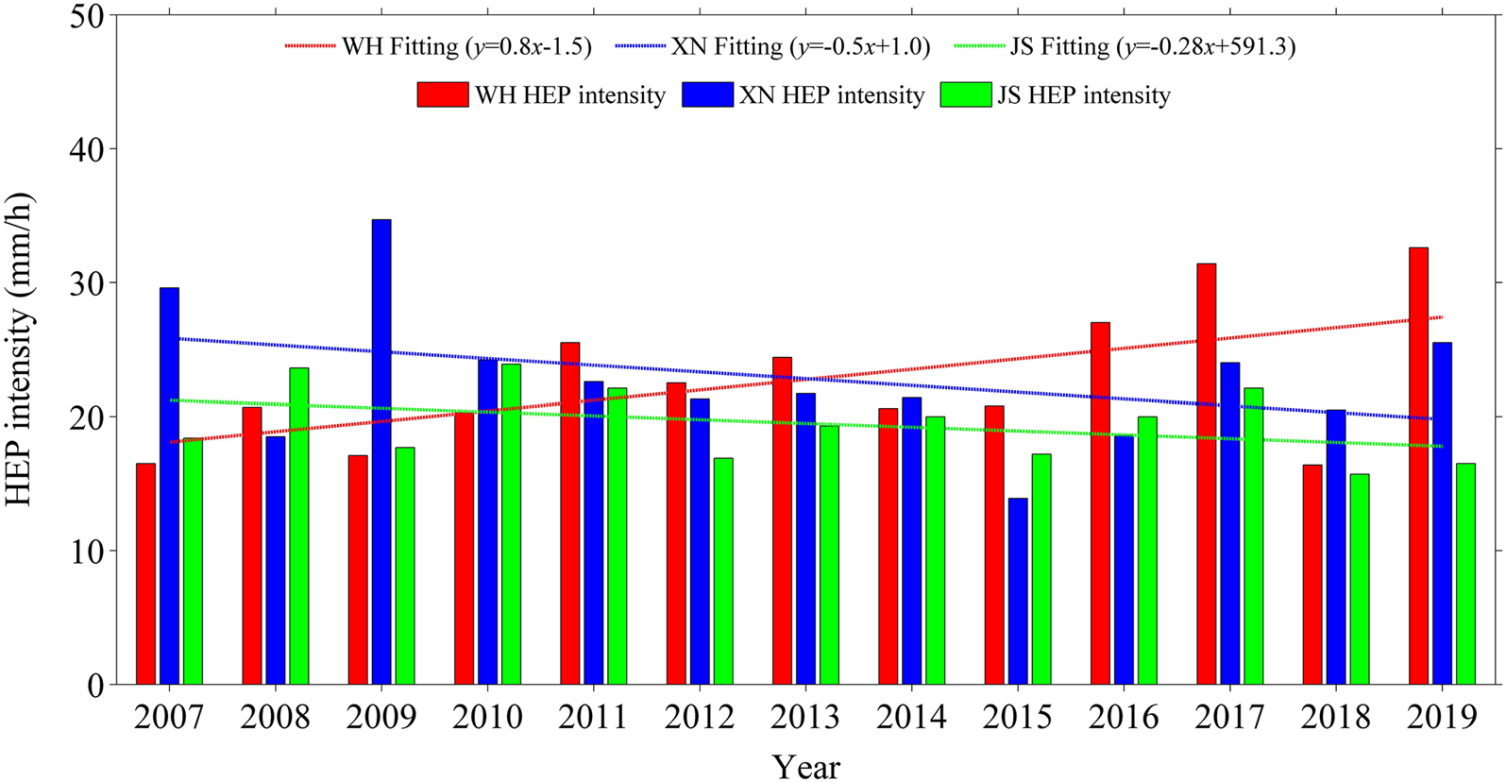
Interannual variations of summer HEP intensity at WH, XN and JS stations during 2007–2019.

Table 1 shows the summer HEP thresholds at WH, XN and JS stations for different percentiles. It can be seen that at different percentiles, all the summer HEP thresholds are ordered as WH > XN > JS, implying that the summer HEP threshold increases with urbanization level. Considering that the sample number decreases with increasing threshold percentile which may bring uncertainty in statistical result, here the HEP threshold at 90th percentile is taken as an example to study the characteristics of summer HEP under different urbanization. Figure 7 presents the summer HEP intensities at WH, XN and JS stations in each year of 2007–2019. Among the three stations, the years of the highest summer HEP intensity at WH is the most, followed by XN, and that at JS is the fewest. This indicates that the summer HEP intensity tends to increase with urbanization level. Moreover, the summer HEP intensity at WH shows a linear increasing trend during 2007–2019, while those at XN and JS present a weak linear decreasing trend. As shown in Fig. 2, the population and urban built-up area in WH is increasing during 2007–2019, but those in XN change little. Obviously, XN has a slow urbanization speed during 2007–2019 and its linear variation trend of summer HEP intensity is close to that of atmospheric background environment monitored at JS. Instead, WH has a rapid urbanization speed and its summer HEP intensity presents a linear increasing trend, indicating that sustainable development of urbanization is beneficial to summer HEP. Therefore, both urbanization level and speed may make impact on summer HEP, and the high the urbanization level and speed, the more conducive for summer HEP.

**Table 1:** Summer HEP thresholds at WH, XN and JS stations for different percentiles.

Station	HEP threshold (mm)					
	90th pct	92th pct	94th pct	95th pct	97th pct	98th pct
WH	13.7	15.9	19.2	24.6	33.1	43.8
XN	12.8	14.0	15.5	21.4	28.5	32.7
JS	11.7	12.1	13.2	19.3	24.1	27.6

**Figure 8:**
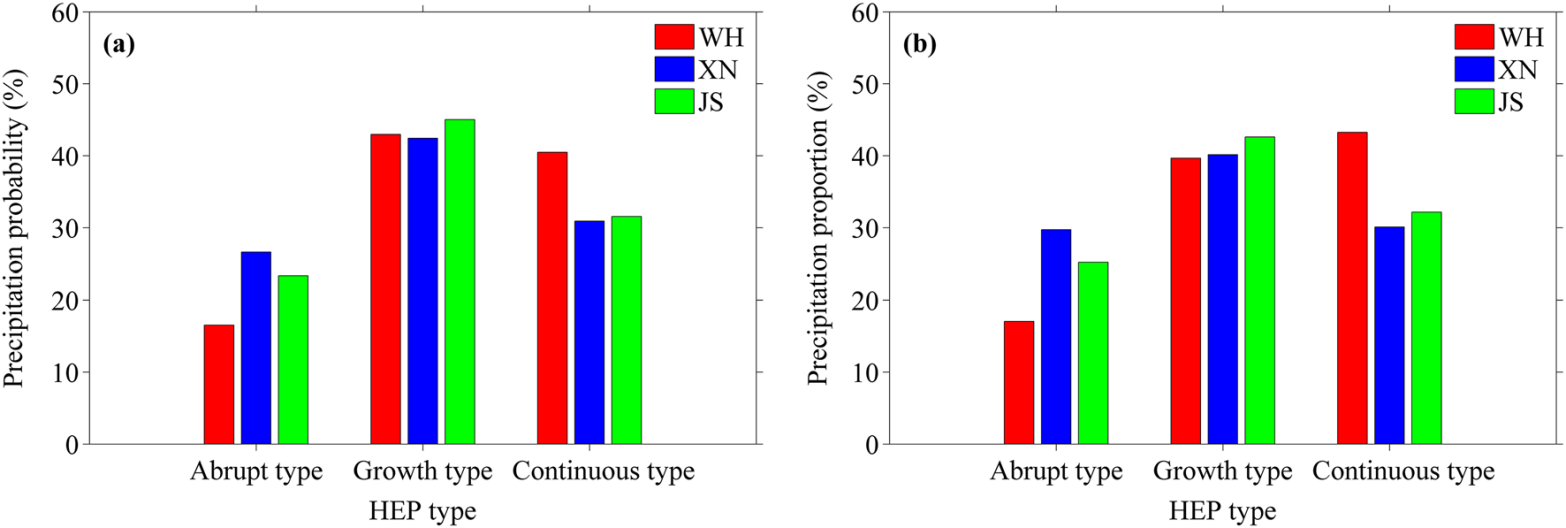
Precipitation (**a**) probability and (**b**) proportion of the three HEP types at WH, XN and JS stations during 2007–2019.

To further study the characteristics of summer HEP, the classification method of Liang and Ding^6^ is adopted to divide summer HEP events into abrupt, growth and continuous types based on the characteristics of earlier 3-h precipitation evolution before the occurrence of HEP, and the classification criteria is shown in Table 2. After that, the precipitation probability and proportion of the three HEP types at WH, XN and JS stations during 2007–2019 are calculated. As shown in Fig. 8a, among the three HEP types, the mean precipitation probability of growth type is the highest (~ 40%), followed by the continuous type (~ 30%), and that of the abrupt type is the lowest (~ 20%). Moreover, the HEP of growth type occurs most at JS (45%) while that of continuous type occurs most at WH (40.5%), and the HEP of abrupt type occurs most at XN (26.6%). Figure 8b shows that the precipitation proportions of the three HEP types are almost the same situation as that for the precipitation probability. Note that compared to XN and JS, the precipitation probability of the continuous type at WH (40.5%) is slightly less than that of the growth type (43%), and the precipitation proportion of the continuous type at WH (43.3%) even surpasses that of the growth type (39.7%). Apparently, the HEP of growth type occurs more frequently than those of continuous and abrupt types regardless of urbanization level, and usually makes main contribution to total precipitation amount of HEP events, but under high urbanization level and speed, the HEP of continuous type tends to increase as well as its contribution to total precipitation amount of HEP events. This is related to the UHIE, because the relatively high temperature in urban atmospheric boundary layer results in much unstable energy accumulation in near surface atmosphere^20^, which may allow the HEP event to last long.

**Table 2:** Classification criteria for the tree types of summer HEP based on the characteristics of earlier 3-h precipitation evolution before the occurrence of HEP.

HEP type	Criteria
Abrupt type	*P-*_*1*_ < 0.5 mm and *P-*_*2*_ < 0.5 mm and *P-*_*3*_ < 0.5 mm
Growth type	(*P-*_*1*_ > *P-*_*2*_ or *P-*_*1*_ > *P-*_*3*_) and at least one (*P-*_*1*_, *P-*_*2*_, *P-*_*3*_) > 0.5 mm but < *P*_*0*_
Continuous type	At least one (*P-*_*1*_, *P-*_*2*_, *P-*_*3*_) > *P*_*0*_

*P-*_*1*_, *P-*_*2*_, *P-*_*3*_ are the precipitation 3 h before the occurrence of HEP, and *P*_*0*_ is the HEP threshold.

## Discussion

**Figure 9:**
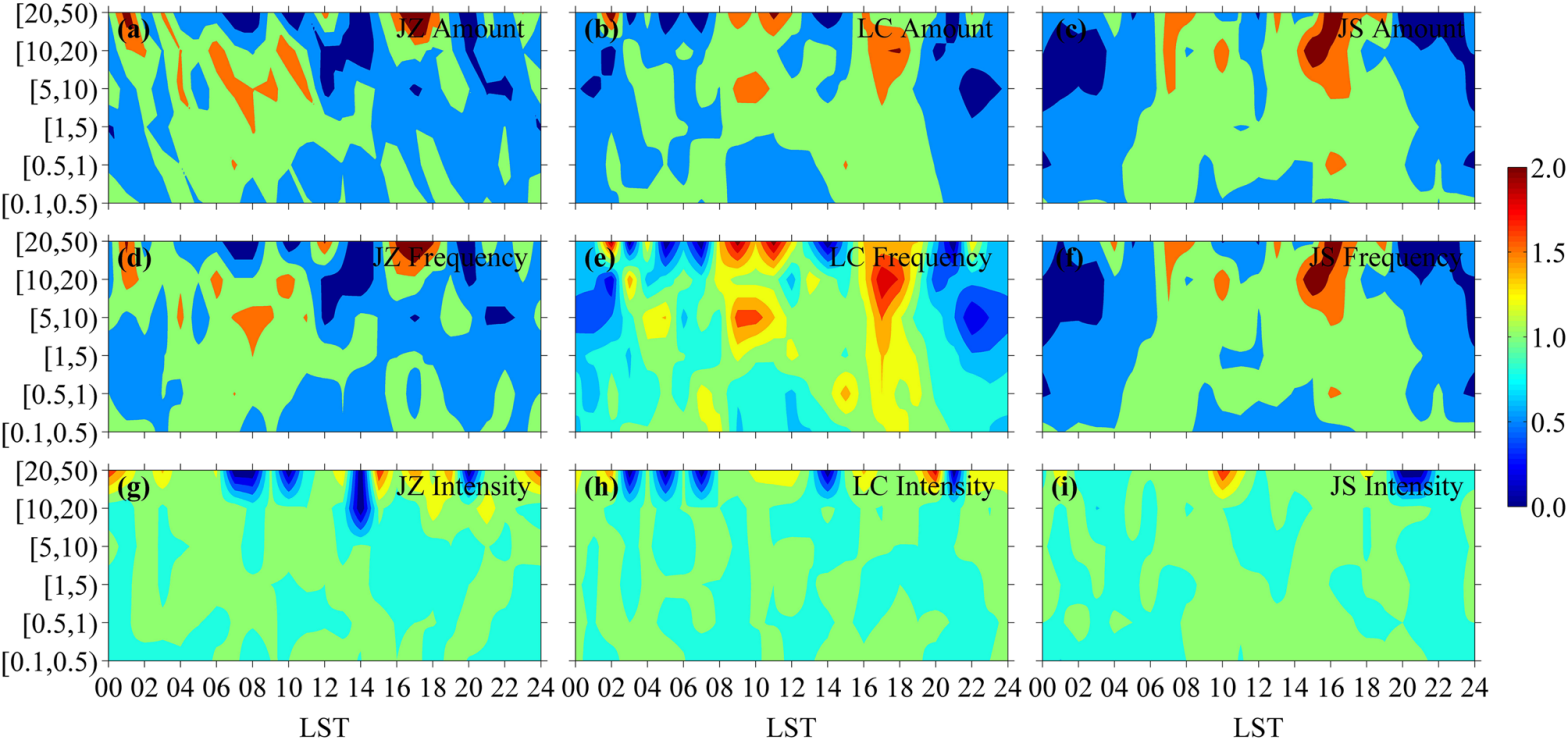
Diurnal variations of precipitation (**a**–**c**) amount, (**d**–**f**) frequency and (**g**–**i**) intensity with dimensionless processing obtained from summer hourly precipitation data of JZ (left), LC (middle) and JS (right) stations during 2007–2019.

**Figure 10:**
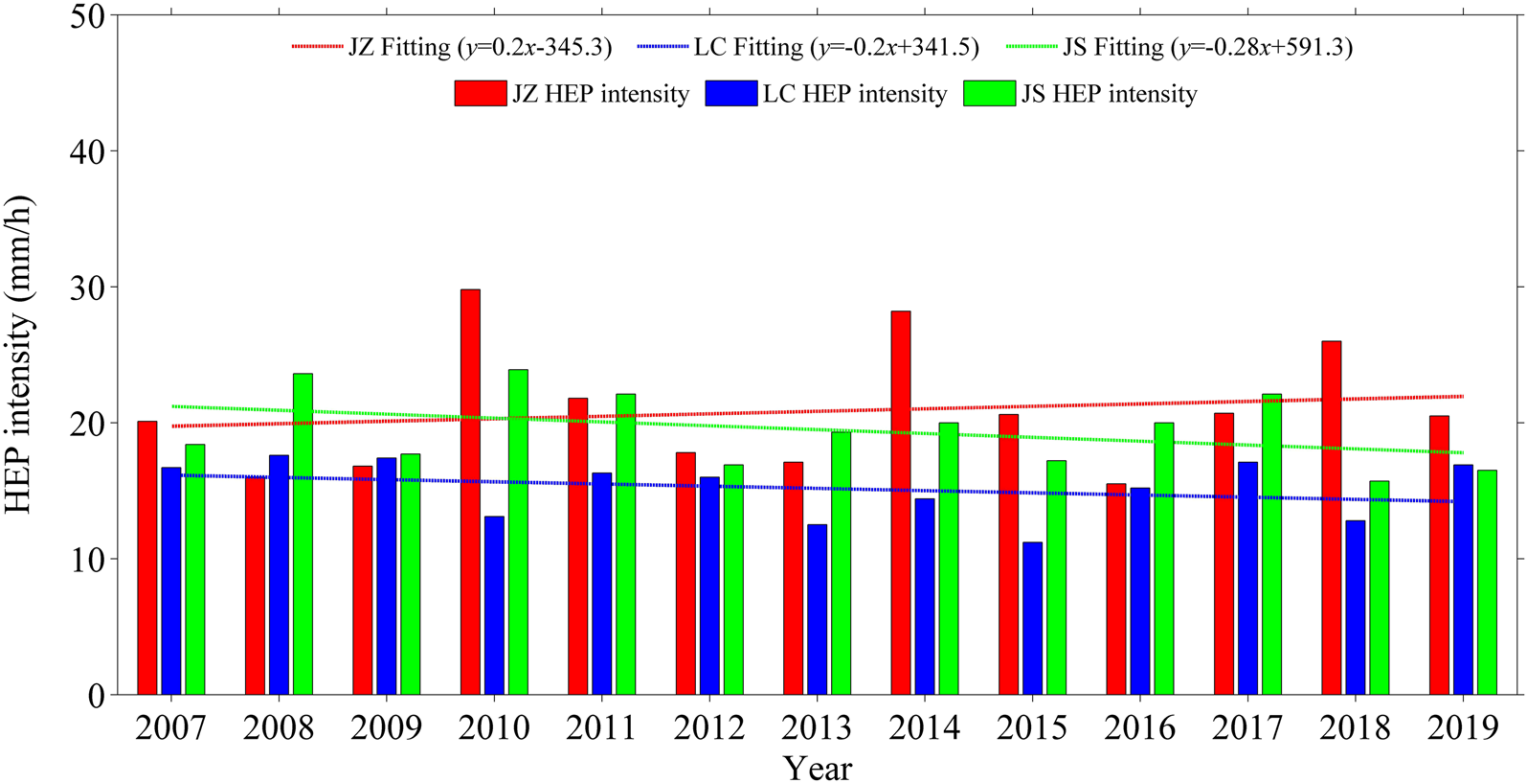
Interannual variations of summer HEP intensity at JZ, LC and JS stations during 2007–2019.

To confirm the above results, the two stations of JZ and LC are also analyzed for comparison. As shown in Figs. 1 and 2, JZ (31.8 m ASL) is in plain with similar terrain to WH (23.6 m ASL), and LC (1074.1 m ASL) is in mountain area with similar terrain to the ABMS of JS (751.4 m ASL), but JZ’s urbanization level is between those of WH and XN, and LC’s urbanization level is between those of XN and JS. It is found that the accumulated precipitation amount and precipitation frequency of summer hourly precipitation are higher at LC than at JZ in most years, but the precipitation intensity is opposite (Figure not shown). Figure 9 presents the diurnal variations of precipitation amount, frequency and intensity with dimensionless processing obtained from summer hourly precipitation data of JZ, LC and JS during 2007–2019. It can be seen that the diurnal variation patterns of precipitation amount and frequency at JZ are more similar to those at WH (Fig. 5), while the diurnal variation patterns of precipitation amount and frequency at LC are more similar to those at JS; also, the diurnal variation pattern becomes complex under high urbanization level. Moreover, Fig. 10 shows that the summer HEP intensity at JZ is also higher than that at LC in most years, and the summer HEP intensity tends to increase at JZ (similar to that at WH) yet decrease at LC (similar to that at JS). Furthermore, the HEP of growth type also occurs more frequently at JZ and LC than those of continuous and abrupt types, and makes main contribution to total precipitation amount of HEP events (Figure not shown). These are consistent with the above results. However, some differences are found in summer hourly precipitation between LC and JS, for example, LC has low urbanization level but its summer HEP intensity is lower than that of JS in most years. As LC is in large mountain area while JS is in small mountain area and the distance between them is about 500 km (Fig. 1), the regional climate difference may make more contribution than urbanization to the difference of summer HEP intensity between LC and JS.

Many studies indicate that urbanization can make impact on regional precipitation^46–48^. This study finds that the precipitation intensity of summer hourly precipitation increases with urbanization level, and the precipitation probability and proportion of hourly precipitation within [20, 50) mm are in the same situation, indicating that urbanization is conducive to the occurrence of hourly strong precipitation, and this is consistent with the conclusion of Liang and Ding^6^, especially our study makes a quantitative supplement to the precipitation grade for the conclusion. Moreover, both summer HEP threshold and intensity increase with urbanization level, and summer HEP intensity also tends to increase when urbanization development is sustainable, implying that high urbanization level and speed is conducive to summer HEP. However, compare to the station with low urbanization level, the precipitation frequency and amount of summer hourly precipitation are lower at the station with high urbanization level. This is also found in the study of Zhang and Chen^49^, and they believe that the combination of water vapor reduction and heat island will cause deeper urban atmospheric boundary layer but reduce convective available potential energy, which then leads to the decrease of summer precipitation in Beijing. Some studies in India, Europe and the United States also suggest that the rapid development of the city reduces the rainfall activity over the city^42,50,51^. These results show that the impact of urbanization on urban precipitation is not simple but complex.

This study also finds that the diurnal variation of summer hourly precipitation and its interannual variation are different at the five stations with different terrain, indicating that besides urbanization the terrain may make impact on urban precipitation^35^. Fu et al.^52^ indicate that high hourly precipitation intensity is found over the city clusters, while the mountainous areas have high frequency and large amount but low intensity of hourly precipitation, which is consistent with the results of this study. On the one hand, terrain effect is critical to the local circulations, through which makes impact on the amount, frequency and intensity of hourly precipitation and their diurnal variations in the city^20,53^. On the other hand, the UHI circulation can reinforce and interact with the mountain-valley circulation^53^. With the UHI-caused temperature gradient between city and mountainous areas, a vertical wind shear appears near the windward slope of mountains^54^, and this is important to initiate and maintain a mesoscale convective system for precipitation. Also, urbanization effect can cause the enhancement of water vapor flux convergence, which plays a role in increasing the precipitation intensity in city^55^. Dou et al.^56^ indicate that UHI intensity is a key factor to the impact of urbanization on urban precipitation, as the high UHI intensity likely leads to the increase of urban precipitation while the low one likely leads to the decrease of urban precipitation. Wang et al.^57^ and Li et al.^58^ suggest that the impact of urbanization on urban precipitation depends on the urbanization stage. In the early stage, the dominant impact of UHIE usually results in precipitation enhancement effect, however, when the city expands to a certain extent, the decrease of water supply caused by the underlying surface and increase of aerosols will enhance the inhibition effect on precipitation. Besides, the influence of underlying surface change and aerosol emission on urban precipitation is related to their respective intensities^59^.

Furthermore, in central China, the summer HEP of growth type occurs more frequently than those of continuous and abrupt types, usually making main contribution to total precipitation amount of summer HEP events, while under high urbanization level and speed, the summer HEP events of continuous type tend to increase as well as its contribution to total precipitation amount of summer HEP events. This is different from the results in the Guangdong, Hong Kong and Macao urban agglomeration obtained by Luo et al.^20^, where the HEP events of abrupt type tend to occur more frequently. The regional climate difference may be partly responsible for this phenomenon, as some studies indicate that urban precipitation is also related to regional climate background to a certain extent^60,61^.

Accordingly, urban precipitation depends on urbanization, terrain and regional climate background, and it is a comprehensive performance of various influencing factors^20,43^. Especially the impact of urbanization on urban precipitation is an overlapping influence of UHIE, underlying surface change, aerosol and other factors, and the increase or decrease of urban precipitation caused by urbanization depends on the comparison of the influence intensity of various factors. Although the numerical weather model can separate the effects of different urbanization factors in theory, how to identify and reasonably evaluate the relative effects of different factors is still a difficult problem. As a result, this study just focuses on the characteristics of summer hourly precipitation under different urbanization level and does not consider the spatial distribution of urban precipitation. Because the spatial distribution of urban precipitation is complex and influenced by the UHIE, local environmental wind field, the terrain and so on^62,63^, more studies are necessary to explore the characteristics of spatial distribution of urban precipitation under different urbanization level, and this is beyond the scope of this study. Additionally, the dynamics mechanism of urban precipitation is complex because it is a comprehensive performance of many influencing factors, especially it is difficult to study the dynamical process of urban precipitation due to the limitation of high temporal and spatial resolution dynamical profiles, and numerical weather model with reasonable representation ability may be a choice to explore this issue.

## Conclusions

By using the summer hourly precipitation data from 2007 to 2019 at four urban stations (WH, JZ, LC and XN) and an ABMS (JS) in central China, this study is to investigate the temporal variation properties of summer precipitation and the characteristics of summer HEP as well as their differences under different urbanization background. The conclusions are drawn as the following.

The summer accumulated precipitation amounts at WH, XN and JS stations present weak linear increasing trend during 2007–2019, with values of 0.61 mm/a, 1.1 mm/a and 2.9 mm/a, respectively. It is the same situation for summer precipitation frequency but opposite for summer precipitation intensity. The higher the urbanization level, the lower the summer accumulated precipitation amount and precipitation frequency, but the higher the summer precipitation intensity. Additionally, high urbanization level is conducive to the occurrence of summer hourly precipitation within [20, 50) mm, yet short-term extremely heavy precipitation greater than or equal to 50 mm is stronger in mountain area due to the effect of terrain.

Diurnal variations of precipitation amount and frequency for summer hourly precipitation within [5, 50) mm have similar pattern at the same station. The pattern at WH is bimodal with two equivalent peaks yet it is unimodal at XN, while at JS the pattern is also bimodal but the main peak evidently higher than the secondary peak. However, precipitation intensity of summer hourly precipitation presents no significant pattern in diurnal variation, but precipitation intensity has larger daily variation range at JS than at WH and XN for hourly precipitation within [20, 50) mm. Moreover, the diurnal variation of summer precipitation intensity at JS presents small interannual difference, reflecting the stability of atmospheric background environment, but those at XN and WH present large interannual difference especially for WH with high urbanization level. These differences in temporal variation of summer hourly precipitation at the three stations are related to urbanization and terrain.

Both high urbanization level and speed are conducive to summer HEP. The higher the urbanization level is, and the higher the summer HEP threshold is, also the summer HEP intensity tends to be higher. Among the three stations, the summer HEP intensity at WH shows a linear increasing trend of 0.8 mm/h per year during 2007–2019, while those at XN and JS present a weak linear decreasing trend, with values of − 0.5 mm/h per year and − 0.28 mm/h per year respectively. This may indicate that sustainable development of urbanization is beneficial to summer HEP, because the urbanization speed of WH is rapid while those of XN and JS are slow or almost zero. In addition, the HEP of growth type occurs more frequently than those of continuous and abrupt types regardless of urbanization level, and usually makes main contribution to total precipitation amount of HEP events. However, under high urbanization level and rapid urbanization speed, the HEP events of continuous type tend to increase as well as its contribution to total precipitation amount of HEP events.

The mechanism of urban precipitation is complex, because it is a comprehensive performance of various influencing factors including urbanization, terrain and regional climate background. Also the impact of urbanization on urban precipitation is an overlapping influence of UHIE, underlying surface change, aerosol and other factors, and the increase or decrease of urban precipitation caused by urbanization depends on the comparison of the influence intensity of various factors. Therefore, how to identify and reasonably evaluate the relative effects of different factors on urban precipitation is a challenge.

## Data Availability

The urban development data including the population and urban built-up area are from the Hubei Provincial Bureau of Statistics (http://tjj.hubei.gov.cn/) and the Urban Construction Year Book (http://www.mohurd.gov.cn/), while the urban boundary data comes from the satellite-based land use category maps of Tsinghua University (http://data.ess.tsinghua.edu.cn), and the station geographic information and summer hourly precipitation data are obtained from the National Meteorological Information Center of China (http://data.cma.cn/).
